# Using enzymatic hydrolyzate as new nitrogen source for L-tryptophan fermentation by E.coli

**DOI:** 10.1080/21655979.2019.1700092

**Published:** 2019-12-27

**Authors:** Da Xu, Zhen Zhang, Ziqiang Liu, Qingyang Xu

**Affiliations:** a National and Local United Engineering Lab of Metabolic Control Fermentation Technology, Tianjin University of Science and Technology, Tianjin, PR China; b Tianjin Engineering Lab of Efficient and Green Amino Acid Manufacture, Tianjin University of Science and Technology, Tianjin, PR China; c College of Biotechnology, Tianjin University of Science and Technology, Tianjin, PR China

**Keywords:** L-tryptophan, *E.coli*, trypsin, hydrolyzate, nitrogen source

## Abstract

This study presents new methods for hydrolyzing bacterial cell in cyclic utilization of waste bacterial cell for L-tryptophan production by fermentation. Using enzymatic hydrolysis of the pre-treated bacterial cells which were collected from an L-tryptophan fermentation broth, trypsin was selected as the optimal protease for hydrolyzing the bacterial cell. The optimum conditions for hydrolysis were determined by the orthogonal test. Hydrolyzate was then dealt with a compound protease to further increase its content of free amino acids. With the optimum conditions of pH = 8, temperature of 37°C, treatment time of 6 h, and E/S of 4%, the final content of free amino acids in the hydrolyzate was 500.61 mg/g. The hydrolyzate and the yeast extract were added to the medium at the proportion of 1:1, which served as an organic nitrogen source for L-tryptophan production by fermentation. The production of L-tryptophan was 53.87 g/L, and the highest biomass was 53.45 g/L. As an organic nitrogen source, this hydrolyzate satisfies the requirements for L-tryptophan production by fermentation.

## Graphical Abstract

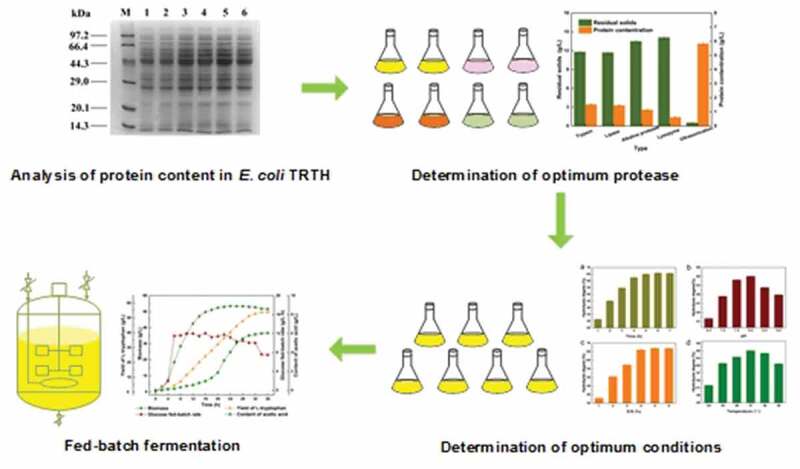

## 1. Introduction

The production of L-tryptophan is continuous innovation, while the costs of production are being reduced. This renders L-tryptophan the fourth most produced amino-acid additive after L-glutamate, L-lysine, and L-threonine [1,2]. L-tryptophan is widely used in the field of food, medicine, feed, and other industries [2–4]. Especially, in the field of feed processing, the demand of L-tryptophan has been increasing recently [5,6]. The market demand for L-tryptophan is also on the rise. L-tryptophan is a product of bulk amino-acid fermentation. Controlling the costs of production and disposal of industrial by-products greatly affects the market competitiveness of products.

Using yeast extract as the main source of organic nitrogen in L-tryptophan production by fermentation has the following deficiencies [7–11]. The composition of yeast extract is complicated, and levels of major nutrients can vary greatly between batches. This may cause fluctuations in the production of L-tryptophan, with some batches having an uncertain acid content. In addition to possessing essential nutrients, yeast extract can also show high levels of impurities such as proteins and pigments [12]. An endotoxin contained in yeast extract is harmful to the bacterial cell, curtailing the upper limit of L-tryptophan production by fermentation [13]. The higher price of yeast extract makes it difficult to maintain lower production costs in bulk fermentation [14].

Microbial fermentation is accompanied by the production of large amount fermentation by-products such as low levels of organic acids, nucleosides, amino acids, and high levels of bacterial proteins. The common bacterial treatment method is to use the bacteria as an organic fertilizer after a simple treatment. This reduces the added value of the product and is a great waste of resources.

Using the appropriate strategy to clean and regenerate the bacteria, produced after fermentation, can greatly reduce the waste of resources and environmental pollution. In order to reuse the waste bacteria and reduce the cost of fermentation, we enzymatically treated the *E. coli* cells left over at the end of the fermentation, and then used these cells for the cultivation of *E. coli*. The abundant amino acids and nucleotides, release from the cells, can serve as a good source of organic nitrogen for tryptophan fermentation. The composition and ratio of the nutrient components fully satisfied the requirements for the growth of *E. coli*. As a source of fermented organic nitrogen, it is advantageous to improve the protein titer of the bacterial cells. Compared with acid-base hydrolysis, using an enzyme to hydrolyze the bacteria is a milder approach, and the obtained hydrolyzate possesses higher nutrient content and is more suitable for use in the fermentation medium. Therefore, several enzymes were assessed to determine the optimal conditions for enzymatic hydrolysis. The components of enzymatic hydrolyzates were compared with those of yeast extract. The proportioning portion partially replaced yeast extract in the fermentation medium to achieve improved recycling of the organic nitrogen source.

Xu et al. studied the effect of sulfuric acid concentration and temperature on the hydrolysis of the waste bacterial cell. The hydrolyzate was added to the fermentation of L-tryptophan production instead of nitrogen source. The L-tryptophan yield and productivity were improved effectively. Moreover, the utilization of waste bacterial cell made the production of L-tryptophan fermented. This is cheaper and is expected to be implemented in industrial production [15]. However, simple hydrolysis of waste bacterial cell by adjusting sulfuric acid concentration and temperature can not make full use of waste bacterial cell. Therefore, we want to hydrolyze the macromolecule proteins of the waste bacterial cell into amino acids and short-chain peptides which are more conducive to the utilization of bacteria by enzymatic hydrolysis, and this may be a new and effective way to improve the utilization rate of waste bacterial cells in L-tryptophan production by fermentation.

## 2. Materials and methods

### 2.1 Microorganisms and materials

The L-tryptophan producer *E. coli* TRTHBPA (*trp*EDCBA+Tet^R^, Δ*tna*, Δ*glt*B, Δ*pta*, Δ*ack*A) used in this study, was generated previously in our laboratory and stored at the Culture Collection of Tianjin University of Science and Technology. The bacteria used for enzymatic hydrolysis were derived from the fermentation broth produced by the above-mentioned strain. In order to select the optimal enzyme for hydrolyzing the bacterial cell, we assessed several enzymes. The essential parameters and optimal conditions for the enzyme activity are shown in Table 1.

**Table 1.** The essential parameters and optimal conditions of enzyme activity.

Enzyme	Manufacturer	Activity(U/g)	Temperature(°C)	pH
Trypsin	Sinopharm Chemical Reagent Co., Ltd.	≥ 50,000	37-38	7.5–8.5
Alcalase	Beijing Solarbio Science & Technology Co., Ltd	≥ 200,000	40-50	9-12
Lipase	Sinopharm Chemical Reagent Co., Ltd.	≥ 3000	36-37	7.0
Muramidase	Sinopharm Chemical Reagent Co., Ltd.	≥ 20,000 U/mg	35	4-6.5

### 2.2 Pretreatment

After centrifugation, the fermentation broth was washed three times with physiological saline solution and diluted into a dry weight of 15 g/L concentration; this was designated as the initial cell suspension. Then, hydrochloric acid was added to the cell suspension at 0.2 mol/L, and heat treatment was conducted for 1 h at 100°C. The initial cell suspension was then subjected to enzymolysis.

### 2.3 Measurement of protein concentration

The supernatant protein concentration was analyzed by a Protein Assay Kit (Nanjing Jiancheng Bioengineering Institute, China) with bicinchoninic acid (BCA) used as standard. For comparison, the values of 100% protein concentration were determined using cells treated by ultrasonication for 15 min, with debris removed by centrifugation at 12,000 g for 15 min at 4°C. The degree of hydrolysis was the percentage of protein concentration in the hydrolyzate supernatant compared with the concentration of 100% protein.

### 2.4 Measurement of solid residues

Cell dry weight was determined by centrifuging the sample, washing cell pellet twice with 0.9% NaCl, and drying it at 90 °C for 24 h [8], the concentration of solid residues is the dry weight of underlayer deposition of 1 ml hydrolyzate.

### 2.5 SDS-page

SDS-PAGE was conducted as described previously [16]: The samples were first boiled at 95°C for 5 min after the addition of 20% (v/v) 5× SDS loading buffer (250 mM Tris-HCl pH6.8, 10% SDS, 0.5% BPB, 50% glycerol, 5% 2-ME). Electrophoresis was then performed in the 0.75 mm-thick SDS-PAGE gel containing 12% polyacrylamide in Tris-glycine running buffer (25 mM Tris, 250 mM glycine, 0.1% SDS) at 80–100 V, and proteins were further visualized by staining with Coomassie R250 solution (0.1% Coomassie R250, 25% isopropanol, 10% acetic acid).

### 2.6 Analysis of amino-acid concentration

The amino-acid analysis of bacterial cell extracts was conducted on an S-433D amino-acid analyzer (Sykam GmbH, Germany) as described previously [17]: Detection was carried out at two wavelengths (570 and 440 nm) according to the manufacturer’s instructions. The separation was performed on a strong acid cation exchange resin with a sulfonic group (PEEK, 4.6 × 150 mm, 7 um). The amino acids were eluted sequentially through the eluent at the rate of 0.45 mL/min at different pH.

### 2.7 Media and culture

The composition of the media and methods of culture have been described previously [18]: A 500 mL baffled flask containing 30 mL seed medium was inoculated with a single colony of E.coli TRJH and cultivated at 36°C, 200 rpm for 12 h. A 30 mL inoculum of this culture was added aseptically to a 5 L seed fermenter (Biotech-2002 Bioprocess controller, Bao Xing, Shanghai, China) containing 3 L seed medium and cultivated at 36°C for 16 h. The pH was adjusted to 7.0 with 25% (v/v) ammonia during the whole cultivation period. The DO level was maintained at approximately 20% saturation by adjusting the agitation and aeration rates.

Fed-batch fermentation was performed in 30-L jar fermenters (Biotech-2002 Bioprecess controller); 30-L jar fermenters containing 18 L production medium were inoculated aseptically with the culture developed in the seed fermenter (10% v/v). In the fermentation process of L-tryptophan, the temperature was maintained at 36°C and the pH was adjusted to 7.0 with 25% (v/v) ammonia. The DO level was maintained at approximately 20% saturation by adjusting the agitation and aeration rates. When the initial glucose was depleted, 800 g/L glucose solution was fed into the fermenter to meet specific experimental requirements.

### 2.8 Analysis of fermentation products

The analysis of fermentation products was described previously [19]: Acetate concentrations were measured using a Bioprofile 300A biochemical analyzer (Nova Biomedical, Waltham, MA, USA).

### 2.9 Statistical analysis

All experiments were conducted in triplicate, and the data were averaged and presented as the mean ± standard deviation. One-way analysis of variance followed by Dunnett’s multiple comparison test was used to determine significant differences. Statistical significance was defined as *p*< 0.05 [19].

## 3. Results and discussion

### 3.1 Analysis of 100% protein content in *E.coli* TRTH

To assess the distribution of the 100% protein of the *E. coli* TRTH strain, we used SDS-PAGE to analyze the samples of the supernatant obtained after ultrasonication.

**Figure 1.**
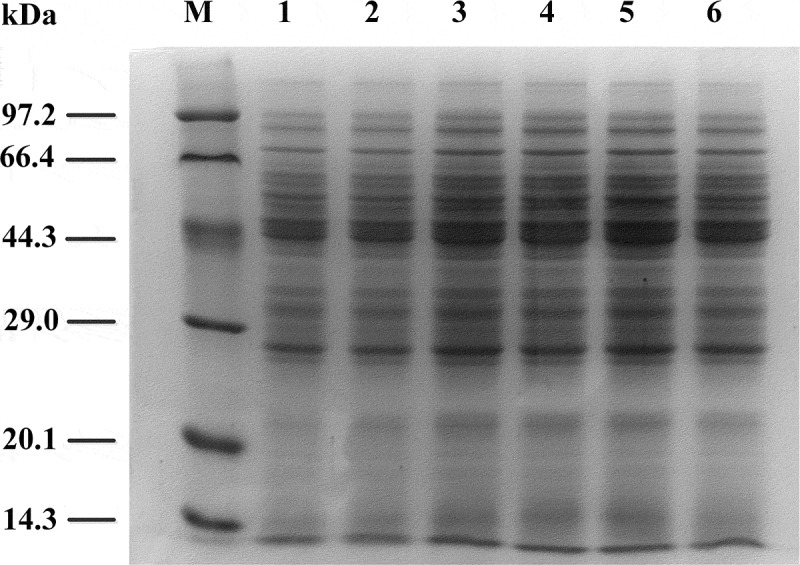
SDS-PAGE analysis of *E. coli* TRTH after ultrasonication. M: Low molecular weight protamine (LMWP) marker. Lanes 1 and 2: 5-μL samples. Lanes 3 and 4: 10-μL samples. Lanes 5 and 6: 15-μL samples.

As shown in Figure 1, when the quantities of loaded samples were 10 and 15 μL, the samples were significantly displayed by SDS-PAGE gel. The relative molecular weight of 100% protein in this strain ranged between 44.3 and 66.4 kDa, and residual protein molecular weight was at approximately 29.0 kDa. The distribution of proteins below 20.1k Da and above 97.2 kDa was very low. There were few small proteins under the minimum molecular weight of 14.3 kDa. The residue concentration was 0.74 g/L, and the protein concentration of the supernatant was 5.83 g/L in samples that have been processed by ultrasonic.

### 3.2 Effect of the four enzymes on enzymolysis of E.coli TRTH

Compared with *Saccharomyces cerevisiae*, the enzyme in *E. coli* can only hydrolyze the peptidoglycan of the cell wall [20,21]; therefore, this enzyme cannot hydrolyze its protein into amino acids, preventing self-hydrolysis. Hydrolysis in *E. coli* can only be obtained by acid, alkali, or proteases [22,23]. Therefore, four different enzymes were selected to hydrolyze the suspension. The best enzyme was determined by comparing the degree of hydrolysis, relative molecular weight of the protein, and solid residue of different hydrolyzates.

**Figure 2.**
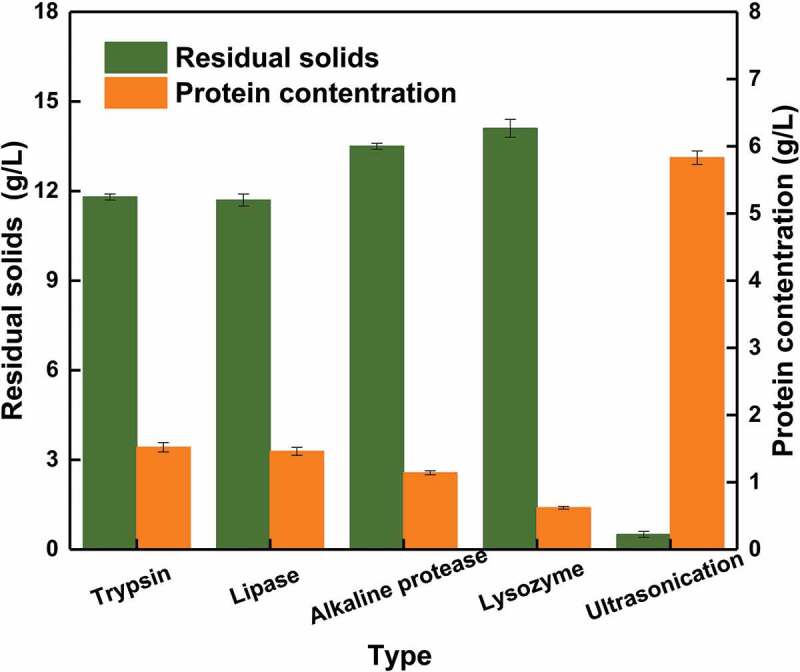
Effect of the four enzymes on protein concentration and residual solids in initial cell suspension.

As shown in Figure 2, the protein concentrations of hydrolyzates treated with the four enzymes were markedly lower than that of the sample treated with ultrasonication. Accordingly, the percentage of solid residues in enzyme-treated samples was much higher than that in samples that have been processed by ultrasonic. Among the four enzymes, trypsin and lipase produced better cell disruption, showing a protein concentration of 1.52 g/L and 1.46 g/L, respectively; solid residue concentration was 11.8 g/L and 11.7 g/L, respectively.

**Figure 3.**
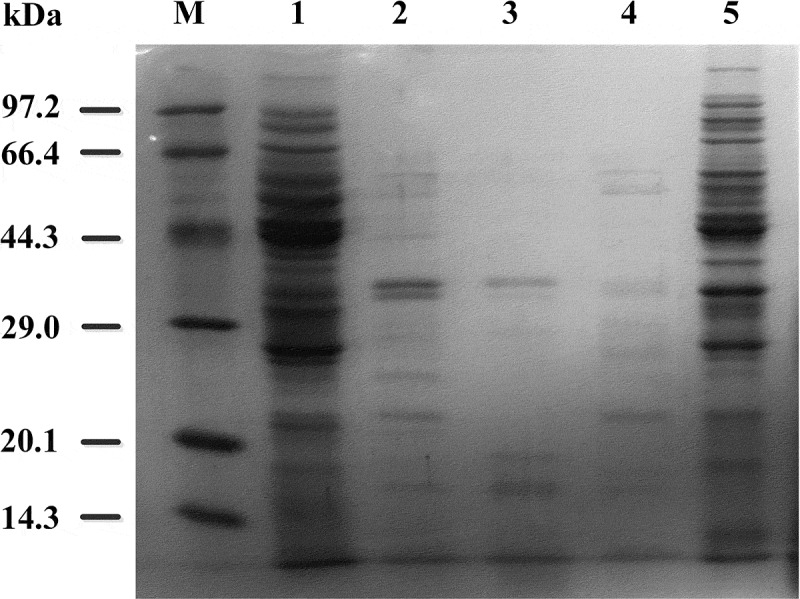
SDS-PAGE analysis of initial cell suspension after treatment with the four enzymes. M: LMWP Marker. 1. Samples having 100% protein content. 2. Samples of hydrolyzate obtained using lipase. 3. Samples of hydrolyzate obtained using trypsin. 4. Samples of hydrolyzate obtained using alkaline protease. 5. Samples of hydrolyzate obtained using lysozyme.

Compared with the samples which have been processed by ultrasonic in Figure 3 (Lane 1), the samples treated with lipase, trypsin, or alkaline protease (Lanes 2, 3 and 4, respectively) showed different degrees of hydrolysis. The proteins in the three enzyme-treated samples rarely distributed between 14.3 kDa and 97.2 kDa.

In summary, although trypsin and lipase were able to hydrolyze the mycoprotein, their ability to release intracellular proteins was weak. This highlights the need to select an economical and effective method for improving the protein concentration in the supernatant before enzymolysis.

### 3.3 Effect of pretreatment on enzymatic hydrolysis

To improve the protein concentration in the supernatant, we used the pre-treatment method described in Section 2.2 to treat the initial cell suspension before enzymolysis. Then, trypsin and lipase were used to hydrolyze this pre-treated cell suspension in order to evaluate the hydrolysis effect.

**Figure 4.**
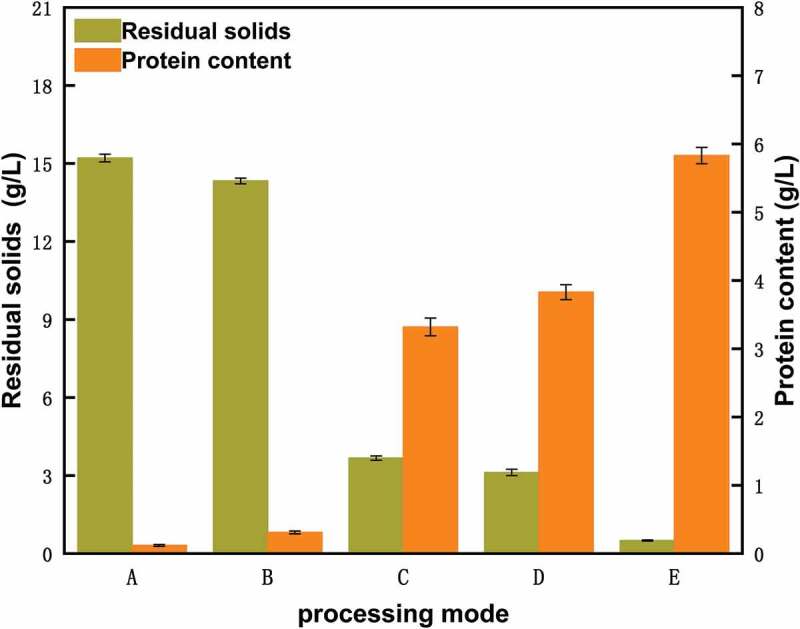
Effect of the supernatant in initial cell suspension (a), pretreated cell suspension (b), hydrolyzate obtained using lipase (c), hydrolyzate obtained using trypsin (d) and ultrasonicated cell suspension (e) on protein concentration and concentration of solid residues.

The concentrations of solid residues before and after pretreatment were 15.21 g/L and 14.33 g/L, respectively. Protein concentrations were 0.15 g/L and 0.11 g/L, respectively. There were no obvious changes in these two parameters. However, the protein concentration in the supernatant of the enzymatic hydrolyzate treated with trypsin was 3.83 g/L and the degree of hydrolysis was 65.7%. The protein concentration in the supernatant of the hydrolyzate treated with lipase was 3.31 g/L and the degree of hydrolysis was 56.8% (Figure 4). Accordingly, most of the bacteria were lysed, and a large number of proteins were dissolved. Using pretreatment altered permeability of the cell membrane, making the selectively permeable cell membrane full permeable. This enabled trypsin and lipase to easily dissolve and hydrolyze the intracellular proteins, achieving protein hydrolyzate. Trypsin was selected as the best enzyme to hydrolyze the bacteria after considering the efficiency of enzymolysis and cost factors.

### 3.4 Optimizing conditions for enzymolysis using trypsin

We used two steps to obtain the complete hydrolysis of mycoprotein in this study. The first step is hydrolyzing the bacteria to release the contents of the cell. The second step is the hydrolysis of the proteins. In this process, the large protein molecules, present in the release cell content, are broken down into short peptides and free amino acids, which can be absorbed directly by bacteria. Before determining the optimal conditions for these processes, we conducted a preliminary experiment to investigate the hydrolysis effect of trypsin.

**Figure 5.**
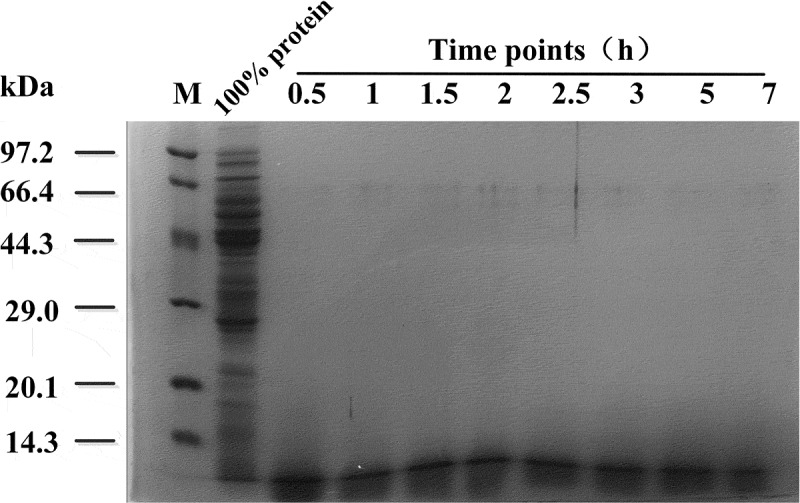
SDS-PAGE analysis of hydrolyzate supernatant obtained using trypsin over different time lengths. M: LMWP Marker.

**Figure 6.**
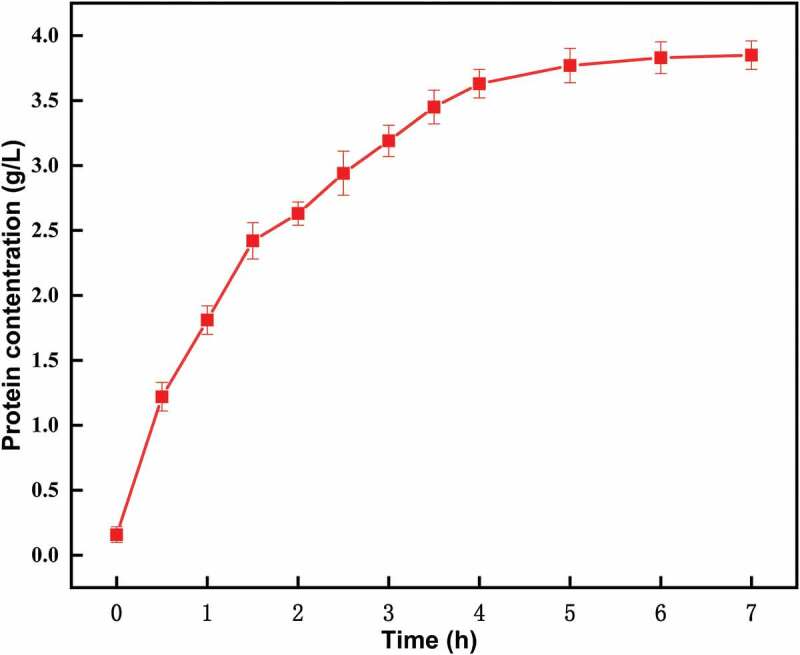
Protein concentration in the supernatant, hydrolyzed by trypsin over different time lengths.

During the process of hydrolysis, the relative molecular weight of hydrolyzate protein at each time point was below 14.3 kDa (Figure 5). However, the protein concentration accumulated at different times. After 5 h, the accumulation rate of protein concentration became slow and then kept constant (Figure 6). The rate-limiting step in trypsin-induced hydrolysis is the release of intracellular proteins. We had previously determined that the optimal time of trypsin treatment in this strain is 5 h, and the evaluation index of the hydrolysis achieved is the degree of hydrolysis.

### 3.5 Parameters affecting the hydrolysis of E.coli TRTH

Parameters affecting the hydrolysis of *E.coli* TRTH include pH, temperature, time of treatment, and the mass ratio of enzyme to the substrate (E/S). These parameters were evaluated and optimized by the orthogonal test.

Optimization of the above parameters to achieve the most efficient way to hydrolysis was determined using single-factor and orthogonal tests, respectively [24,25]. Parameters affecting hydrolysis, which are the pH, temperature, time or treatment, and the mass ratio of the enzyme to the substrate (E/S), were selected as independent variables. The orthogonal test was conducted using the L9 (3^4^) orthogonal table, and the degree of hydrolysis was the dependent variable.

**Figure 7.**
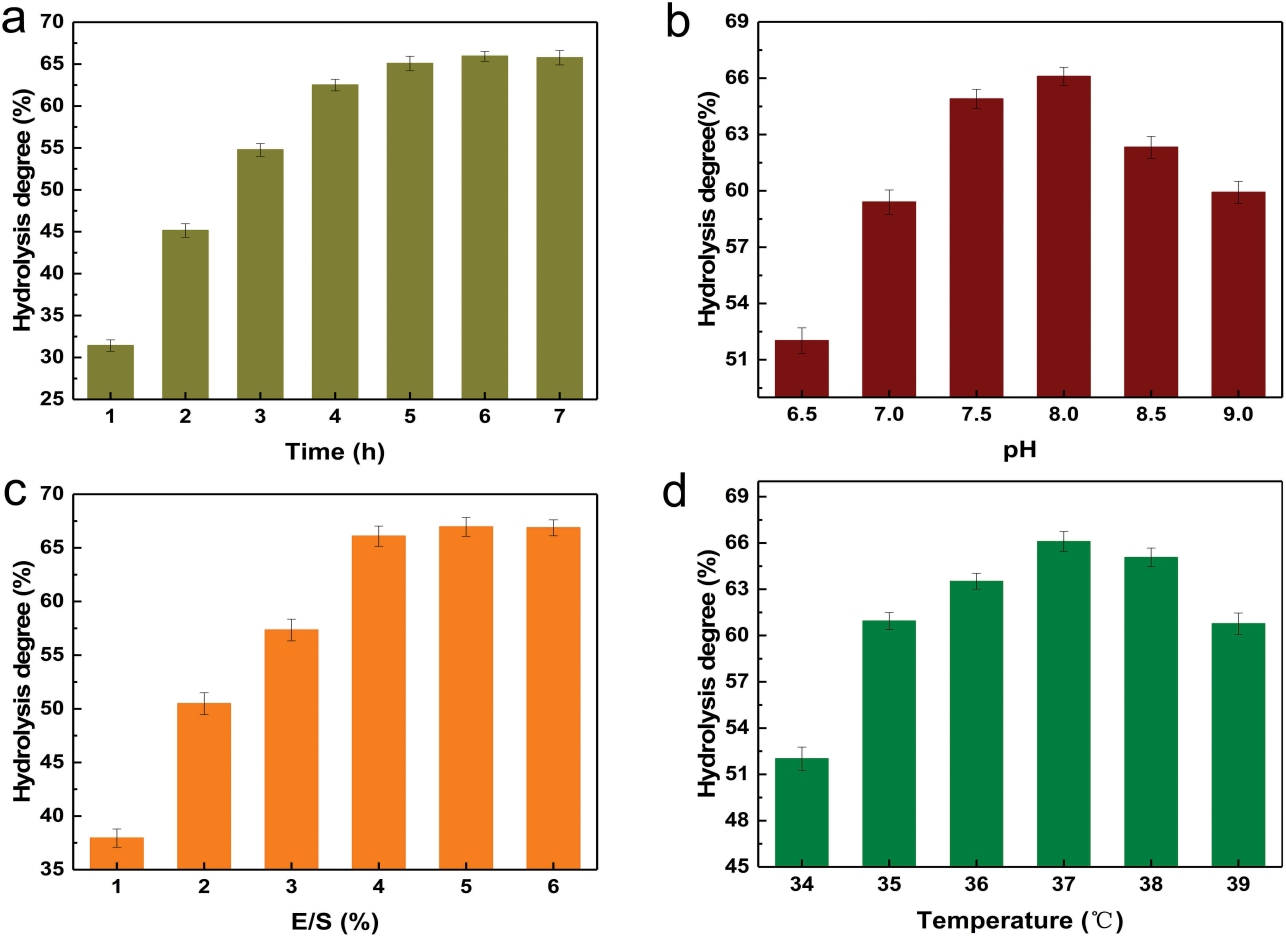
Effects of pH (a), temperature (b), time of treatment (c), and E/S (d) on the degree of hydrolysis.

Figure 7 summarizes the suitable range of each factor. The results of the orthogonal are shown in Table 2. The range analysis indicated that E/S exerted the most influence on the degree of hydrolysis compared with those of pH, temperature, and time of treatment (Table 3). The best combination of conditions was A_2_B_2_C_3_D_2_; using this, we determined the optimal conditions for hydrolysis via trypsin to be at pH = 8, temperature of 37°C, treatment time of 4 h, and E/S of 4% (Table 3). The hydrolysis degree of bacteria treated with trypsin was 66.09% under these optimal conditions.

**Table 2.** The table of orthogonal test factor level.

Level	pH	Temperature(°C)	time(h)	E/S(%)
1	7.5	36	4	3
2	8.0	37	5	4
3	8.5	38	6	5

**Table 3.** Analysis results of orthogonal test.

Number	Factor				
	A: pH	B: Temperature(°C)	C: Time(h)	D: E/S (%)	Hydrolysis degree (%)
1	7.5	36	4	3	57.30
2	7.5	37	5	4	64.89
3	7.5	38	6	5	63.90
4	8.0	36	5	5	63.51
5	8.0	37	6	3	62.14
6	8.0	38	4	4	64.54
7	8.5	36	6	4	62.61
8	8.5	37	4	5	62.32
9	8.5	38	5	3	57.86
K_1_	62.030	61.140	61.387	59.100	
K_2_	63.397	63.117	62.087	64.013	
K_3_	60.930	62.100	62.883	63.243	
R^3^	2.467	1.977	1.496	4.913

### 3.6 Analysis of free amino-acid content

The protein in the hydrolyzate consisted of low molecular weight polypeptides and oligopeptides. The source of organic nitrogen needs to include slow nitrogen sources, as well as products of protein degradation that can be absorbed directly, such as free amino acids. Amino acids can be directly utilized by bacteria via transamination, which is beneficial for the growth of bacteria and shortens the growth stagnation period. To increase the concentration of free amino acids in the hydrolyzates obtained by enzyme treatment, we used a protease complex to hydrolyze the hydrolyzates further. The active enzymes in this complex are aminopeptidase and carboxypeptidase, which can dissociate amino acids from the N-terminus and carboxyl terminus of polypeptide chain one by one.

**Figure 8.**
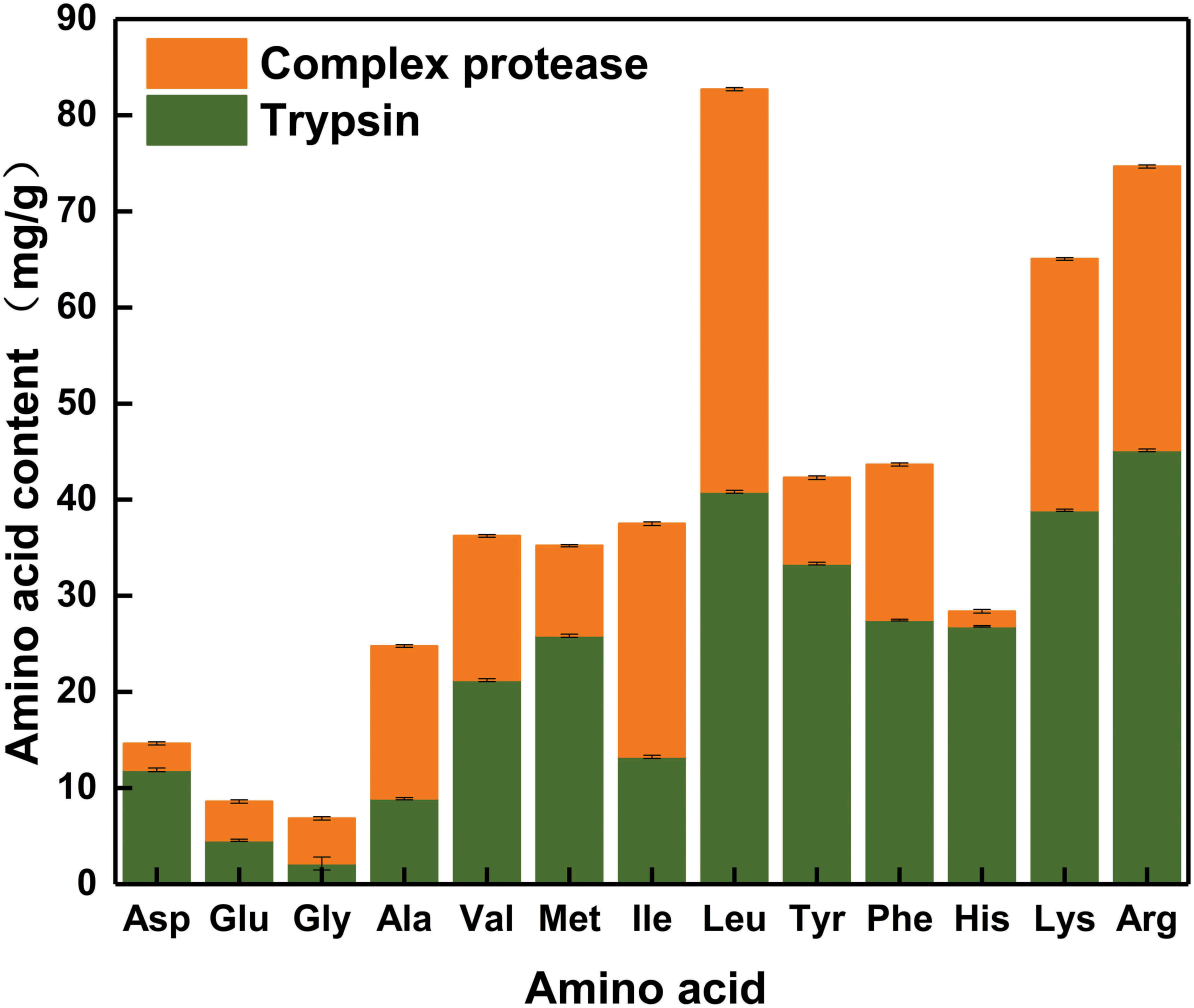
Effect of protease complex on the concentration of free amino acids.

As shown in Figure 8, there were 14 free amino acids in the hydrolyzate obtained using trypsin, as detected by an amino-acid automatic analyzer. Amino acids such as Arg, Lys, and Leu were the commonest amino acids in the trypsin hydrolyzate. After hydrolysis with compound protease, the contents of free amino acids in the supernatant increased to varying degrees.

The total free amino-acid content in yeast extract was 312.69 mg/g, and the total free amino acid in the hydrolyzate was 500.61 mg/g, which is 1.6 times the total amount in yeast extract. Glutamate, alanine, and leucine were the main amino acids in yeast, accounting for 18.6%, 13.1%, and 9.8% of total content. In contrast, the major amino acids in the enzymatic hydrolyzate were leucine, lysine, and arginine, accounting for 16.5%, 14.9%, and 13% of total content (Table 4). Lysine and arginine are essential for the growth of *E.coli* and exert positive effects on the inhibition of acetic acid accumulation in L-tryptophan production by fermentation.

**Table 4.** Comparison of amino acids in yeast extract, trypsin hydrolyzates, and complex protease hydrolyzates.

Amino acid	Yeast extract (mg/g) ^a^	Trypsin hydrolyzates (mg/g) ^b^	Complex protease hydrolyzates (mg/g) ^b^
Asparagine	16.10 ± 0.08	11.89 ± 0.08	14.64 ± 0.09
Tyrosine	14.20 ± 0.07	33.36 ± 0.14	42.30 ± 0.11
Histidine	5.50 ± 0.06	26.82 ± 0.08	28.39 ± 0.15
Proline	8.90 ± 0.05	-	-
Glycine	11.10 ± 0.10	2.15 ± 0.04	6.86 ± 0.04
Methionine	12.00 ± 0.09	25.85 ± 0.10	35.22 ± 0.12
Phenylalanine	12.20 ± 0.11	27.48 ± 0.09	43.66 ± 0.16
Serine	14.60 ± 0.13	-	-
Isoleucine	14.80 ± 0.12	13.25 ± 0.08	37.50 ± 0.14
Lysine	15.20 ± 0.11	38.90 ± 0.11	65.05 ± 0.15
Threonine	15.09 ± 0.14	-	-
Arginine	21.00 ± 0.16	45.15 ± 0.14	74.67 ± 0.18
Valine	22.10 ± 0.16	21.23 ± 0.05	36.23 ± 0.18
Leucine	30.60 ± 0.18	40.82 ± 0.011	82.71 ± 0.16
Alanine	41.10 ± 0.12	8.90 ± 0.08	24.78 ± 0.11
Glutamic	58.20 ± 0.15	4.56 ± 0.09	8.60 ± 0.08
Total	312.69	300.36	500.61

^a^ The proportion of amino acids to yeast extract

^b^ The proportion of amino acids to dry bacteria

### 3.7. Using hydrolyzate instead of organic nitrogen source in L-tryptophan production by fermentation

As a source of organic nitrogen in the medium, hydrolyzate accounted for 50% of total organic nitrogen, and other nutrients remained unchanged.

**Figure 9.**
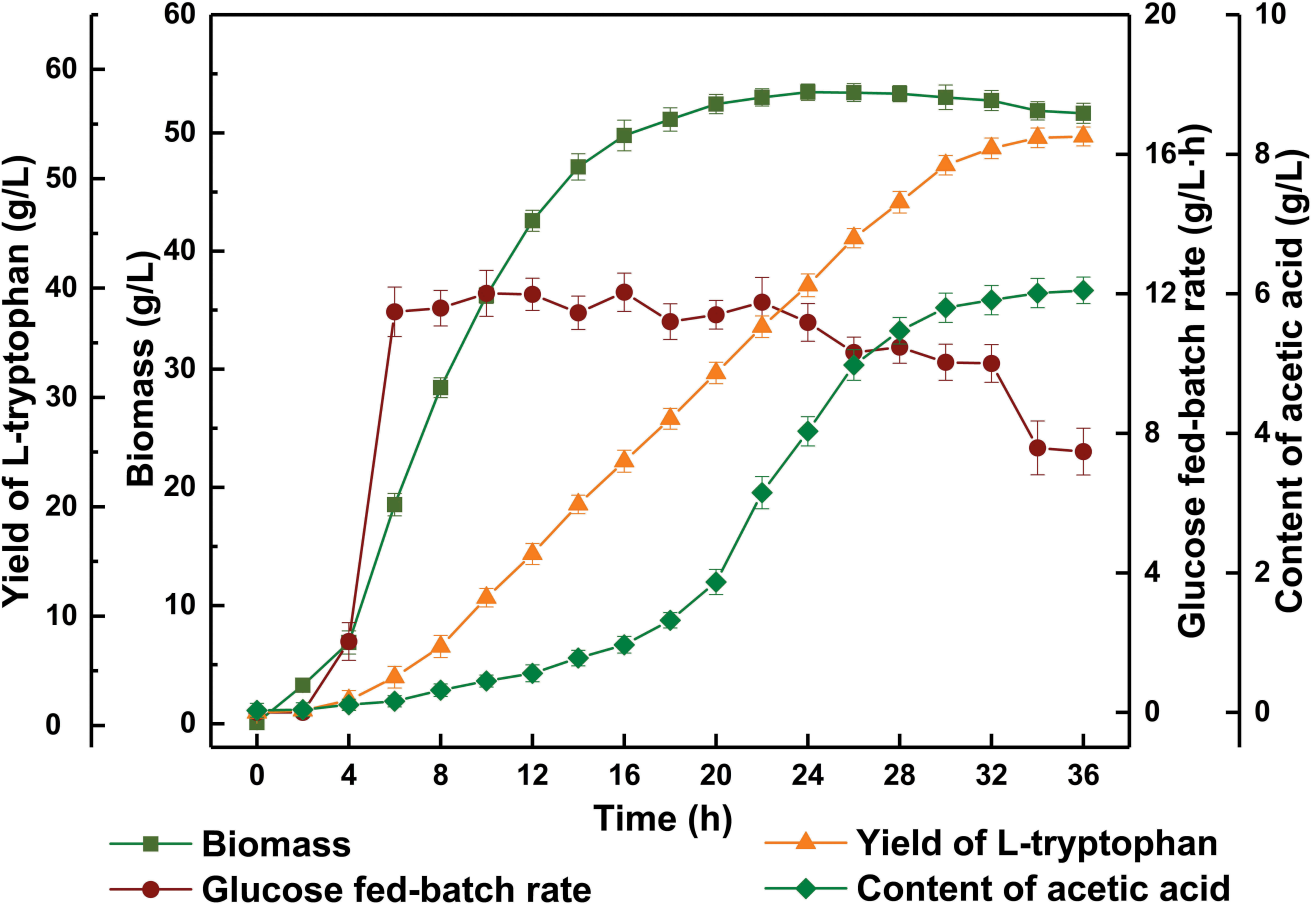
Effect of using hydrolyzate as partial source of organic nitrogen in L-tryptophan fermentation.

The rate of glucose in the fermentation period was always maintained at 10–12 g/L·h. The biomass of the bacteria increased rapidly at first, stabilized after 20 h and stopped growing until the end of fermentation. At 30 h, the bacteria began to decline, and the highest biomass was 53.45 g/L. The production rate of L-tryptophan from 8 h to 30 h was 1.5–2.5 g/L·h, which declined rapidly after 30 h; the final yield was 53.87 g/L. Concurrently, the content of acetic acid, the main by-product of fermentation, reached 5.80 g/L, which affected bacterial viability (Figure 9).

Compared with the fermentation results obtained by Xu et al. [15,26], the final production of L-tryptophan has increased. The highest biomass was at the same levels, indicating that our approach satisfied the current requirements for the production of L-tryptophan by fermentation. However, there were still some deficiencies in this study. These were the high concentration of acetic acidic lead to a shortened period of L-tryptophan production and low biomass. These issues were caused by insufficient content of the slow nitrogen source in the substituted medium. This caused a shortage of nutrients for the bacteria in the middle stage and later stage of fermentation. If we could solve this problem, the fermentation time would be extended and the final production of L-tryptophan would be higher. Moreover, because of the lack of enzymes, we could not possess more kinds of enzymes to hydrolyze the bacterial cell, there may be more suitable enzymes for this study.

## 4. Conclusions

Here, we present a pre-treatment step to improve the release of bacterial protein and the best protease for the hydrolysis of this strain. The optimal treatment conditions were as follows: pH = 8, temperature of 37°C, treatment time of 6 h, and E/S of 4%. The hydrolyzate was hydrolyzed at the second step using aminopeptidase and carboxypeptidase contained in a protease complex. The final free amino-acid content was 500.61 mg/g, which was 1.6 times the total amino-acid content of yeast extract. The content of free amino acids in the hydrolyzate has improved further. After obtaining the hydrolyzate, it was mixed with yeast extract using the same proportions as those used for organic nitrogen source in L-tryptophan production by fermentation. The final fermentation yield of L-tryptophan was 53.87 g/L, the biomass was 53.45 g/L, and the content of the byproduct acetic acid was 5.80 g/L. This hydrolyzate satisfied the requirements for organic nitrogen sources in the production of L-tryptophan.

## Research highlights

- reduce the cost of L-tryptophan production and the environmental pollution of fermentation
- recycle the waste bacterial cell as raw materials for fermentation
- provide a direction for further research on the pollution treatment of microbial fermentation industry.
